# Development and validation of a haematuria cancer risk score to identify patients at risk of harbouring cancer

**DOI:** 10.1111/joim.12868

**Published:** 2019-01-04

**Authors:** W. S. Tan, A. Ahmad, A. Feber, H. Mostafid, J. Cresswell, C. D. Fankhauser, S. Waisbrod, T. Hermanns, P. Sasieni, J. D. Kelly, DETECT I trial collaborators, P Khetrapal, H Baker, AN Sridhar, BW Lamb, F Ocampo, H McBain, K Baillie, K Middleton, D Watson, H Knight, S Maher, A Rane, B Pathmanathan, A Harmathova, G Hellawell, S Pelluri, J Pati, A Cossons, C Scott, S Madaan, S Bradfield, N Wakeford, A Dann, J Cook, M Cornwell, R Mills, S Thomas, S Reyner, G Vallejera, P Adeniran, S Masood, N Whotton, K Dent, S Pearson, J Hatton, M Newton, E Heeney, K Green, S Evans, M Rogers, K Gupwell, S Whiteley, A Brown, J McGrath, N Lunt, P Hill, A Sinclair, A Paredes‐Guerra, B Holbrook, E Ong, H Wardle, D Wilson, A Bayles, R Fennelly, M Tribbeck, K Ames, M Davies, JA Taylor, E Edmunds, J Moore, S Mckinley, T Nolan, A Speed, A Tunnicliff, G Fossey, A Williams, M George, I Hutchins, R Einosas, A Richards, A Henderson, B Appleby, L Kehoe, L Gladwell, S Drakeley, JA Davies, R Krishnan, H Roberts, C Main, S Jain, J Dumville, N Wilkinson, J Taylor, F Thomas, K Goulden, C Vinod, E Green, C Waymont, J Rogers, A Grant, V Carter, H Heap, C Lomas, P Cooke, L Scarratt, T Hodgkiss, D Johnstone, J Johnson, J Allsop, J Rothwell, K Connolly, J Cherian, S Ridgway, M Coulding, H Savill, J Mccormick, M Clark, G Collins, K Jewers, S Keith, G Bowen, J Hargreaves, K Riley, S Srirangam, A Rees, S Williams, S Dukes, A Goffe, L Dawson, R Mistry, J Chadwick, S Cocks, R Hull, A Loftus, Y Baird, S Moore, S Greenslade, J Margalef, I Chadbourn, M Harris, J Hicks, P Clitheroe, S Connolly, S Hodgkinson, H Haydock, A Sinclair, E Storr, L Cogley, S Natale, W Lovegrove, K Slack, D Nash, K Smith, J Walsh, AM Guerdette, M Hill, D Payne, B Taylor, E Sinclair, M Perry, M Debbarma, D Hewitt, R Sriram, A Power, J Cannon, L Devereaux, A Thompson, K Atkinson, L Royle, J Madine, K MacLean, R Sarpong, C Brew‐Graves, N Williams

**Affiliations:** ^1^ Division of Surgery & Interventional Science University College London London UK; ^2^ Department of Urology University College London Hospital London UK; ^3^ Cancer Intelligence Cancer Research UK London UK; ^4^ UCL Cancer Institute London UK; ^5^ Department of Urology Royal Surrey County Hospital Guildford UK; ^6^ Department of Urology James Cook University Hospital Middlesbrough UK; ^7^ Department of Urology University Hospital Zurich University of Zurich Zurich Switzerland; ^8^ Faculty of Life Sciences & Medicine School of Cancer & Pharmaceutical Sciences Innovation Hub Guys Cancer Centre Guys Hospital King's College London London UK

**Keywords:** bladder cancer, detection, haematuria, nomogram, predict, urinary tract cancer

## Abstract

**Background:**

A lack of consensus exists amongst national guidelines regarding who should be investigated for haematuria. Type of haematuria and age‐specific thresholds are frequently used to guide referral for the investigation of haematuria.

**Objectives:**

To develop and externally validate the haematuria cancer risk score (HCRS) to improve patient selection for the investigation of haematuria.

**Methods:**

Development cohort comprise of 3539 prospectively recruited patients recruited at 40 UK hospitals (DETECT 1; ClinicalTrials.gov: NCT02676180) and validation cohort comprise of 656 Swiss patients. All patients were aged >18 years and referred to hospital for the evaluation of visible and nonvisible haematuria. Sensitivity and specificity of the HCRS in the validation cohort were derived from a cut‐off identified from the discovery cohort.

**Results:**

Patient age, gender, type of haematuria and smoking history were used to develop the HCRS. HCRS validation achieves good discrimination (AUC 0.835; 95% CI: 0.789–0.880) and calibration (calibration slope = 1.215) with no significant overfitting (*P* = 0.151). The HCRS detected 11.4% (*n* = 8) more cancers which would be missed by UK National Institute for Health and Clinical Excellence guidelines. The American Urological Association guidelines would identify all cancers with a specificity of 12.6% compared to 30.5% achieved by the HCRS. All patients with upper tract cancers would have been identified.

**Conclusion:**

The HCRS offers good discriminatory accuracy which is superior to existing guidelines. The simplicity of the model would facilitate adoption and improve patient and physician decision‐making.

## Introduction

The decision to guide who should have investigations following a presentation of haematuria varies between guidelines 1. Recommendations by the National Institute for Health and Care Excellence (NICE) suggest that patients aged ≥45 years with visible (VH) and ≥60 years with nonvisible haematuria (NVH) with either dysuria or a raised white cell count on blood test should be urgently investigated 2. Nonurgent referral can be considered for patients ≥60 years with recurrent or persistent unexplained NVH 2. In contrast, guidance from the American Urology Association (AUA) is that all patients with VH and patients with NVH aged ≥35 years should have diagnostic tests although patients younger than 35 years may be referred for cystoscopy at the discretion of the clinician 3.

Consistent across guidelines is the use of age‐specific thresholds to guide referral for the investigation of VH and NVH as increasing age is an established risk factor for bladder cancer. Adopting arbitrary thresholds will invariably result in an increased likelihood of missed cancers as well as over investigation of cases unlikely to harbour malignancy. We have previously reported that 3.5% of patients presenting with VH and 1.0% of patients with NVH have a diagnosis of malignancy despite not meeting the age threshold set out in NICE guidance 4.

Predictive and prognostic tools using statistical models have been developed in the form of nomograms enabling individual patient‐specific risk estimation 5. Nomograms often include multiple parameters with the advantage to outperform specific individual variables. Whilst numerous prognostic nomograms have been developed for bladder cancer, there is only one risk score reported for the prediction of a diagnosis of cancer in patients presenting with NVH 6, 7, 8.

In this study, we report the development and external validation of a haematuria cancer risk score (HCRS) for the prediction of cancer to enable both patients and physicians to easily assess cancer risk following a presentation of haematuria. The advantage of a risk assessment approach over the application of arbitrary age thresholds allows a more individualized approach with the aim to improve detection of cancer and reduce the need for the investigations in patients unlikely to have malignancy.

## Material and methods

### Study design and population

Both the development and validation cohort comprise patients who were referred to secondary care following a presentation of haematuria. NVH was defined as urine dipstick of ≥1+ of blood on ≥2 occasions in the discovery cohort 9. NVH was defined by either haematuria on urine dip stick or urine microscopy in the validation cohort due to the absence of haematuria guidelines in Switzerland and the variation in physician practice patterns. Patients in the development cohort were prospectively recruited between March 2016 and June 2017 at 40 UK hospitals whilst the external cohort consist of patients who were retrospectively identified as having haematuria investigations between 2011 and 2017 from the Department of Urology, University of Zurich. All patients were ≥18 years and were referred from primary care to secondary care following a presentation of haematuria in the community. Study design and patient eligibility criteria of the development cohort have been previously described 10.

All patients had no previous history of a bladder cancer diagnosis and evaluation comprised of medical history and clinical examination. Patient demographics, gender, ethnicity, smoking history and occupation were recorded. Occupational risk factor was defined as patients working as gardener, painter, hairdresser/barber, textile worker or metals factory worker 11. Cystoscopy and upper tract imaging were performed for all patients. Where bladder cancer was suspected, patients had a transurethral resection of bladder tumour (TURBT) or bladder biopsy under general anaesthesia. Bladder cancers were defined as urothelial cell carcinoma and other bladder cancer variants confirmed on histology. Upper tract cancers were also confirmed on histology and classified to either upper tract urothelial cancer or renal cell cancers.

The development cohort of the study received ethical approval by Health Research Authority‐North West Liverpool Central Research Ethics Committee on March 2016 (IRAS project ID: 179245, REC reference: 16/NW/0150). The validation cohort received ethical approval by the Cantonal Ethics Committee of Zurich (STV KEK‐ZH BASEC‐Nr. 2016‐00158).

### Development and validation of a novel haematuria cancer risk score and statistical analysis

Univariate logistic regression was used to determine an association between individual variables and bladder cancer. The outcome was bladder cancer which was defined as Yes = 1 versus No = 0. All cases were used for estimating odds ratios. Age (years) was analysed as a continuous variable whilst gender (0 = female, 1 = male), type of haematuria (0 = NVH, 1 = VH), smoking history (0 = nonsmoker, 1 = ex‐smoker, 2 = current smoker, 3 = missing) and ethnicity (0 = White, 1 = non‐White, 2 = missing) as categorical variables. Multivariate logistic regression model was performed with patient's age, gender, type of haematuria and smoking were used as the final predictors for the diagnosis of bladder cancer (0 = No vs 1 = Yes).

A novel HCRS was developed as the linear predictor of the fitted multivariate logistic regression in the derivation data set and fitted as a single predictor to the validation data set. To assess the performance of the HCRS, the area under the curve (AUC) was used as a measure of discrimination. The lower and upper 95% confidence interval (CI) of the AUC were computed as defined by DeLong *et al*. 12. The Venkatraman's test for two unpaired receiver operating curves (ROC) was performed using 2000 resampling to test the null hypothesis that the true difference in AUC is equal to 0 13. External validation was then performed using the Swiss patient cohort. The prediction accuracy of the HCRS was evaluated by the calibration slope in the validation data set.

All statistical analyses were performed with R (R Foundation for Statistical Computing; version 3.4.3) 14. All applied tests were two‐sided, and a *P*‐value < 0.05 was accepted as statistically significant. No *P*‐value adjustment was performed for multiple comparisons. The development cohort of this study was registered with ClinicalTrials.gov: NCT02676180.

## Results

### Patient demographics of the development and validation cohort

A total of 3539 and 656 patients were used in the development and validation cohort, respectively. Descriptive patient characteristics of both study populations are shown in Table 1. Univariate logistic regression analysis reports that older patients (*P* < 0.001), patients with VH (*P* < 0.001), male patients (*P* < 0.001), White patients (*P* = 0.004) and patients with a smoking history (*P* < 0.001) were significantly more likely to have a bladder cancer diagnosis. In the development cohort, 285 patients (8.1%) had a diagnosis of bladder cancer and 69 patients (10.7%) had a diagnosis of bladder cancer in the validation cohort. Occupational risk factor was not associated with the diagnosis of bladder cancer (*P* = 0.8). Distribution of patient age stratified by smoking history and diagnosis of cancer is described using a box plot in Figure S1. Bladder cancer histopathological characteristics are shown in Table S1.

**Table 1:** Patient demographics of the development and validation cohorts

Variables	Development cohort (*n* = 3539)	Validation cohort (*n* = 656)
Age (median, IQR) [range]	68 (57, 76) [23–96]^a^	57 (47, 68) [18–89]^b^
Haematuria, *n* (%):		
Visible	2296 (64.9)	322 (49.1)
Nonvisible	1243 (35.1)	334 (50.9)
Gender, *n* (%):		
Male	2098 (59.3)	504 (76.8)
Female	1441 (40.7)	152 (23.2)
Ethnicity, *n* (%):		
White	2977 (93.8)	
Non‐White	196 (6.2)	
Smoking history, *n* (%):		
Nonsmoker	1519 (44.6)	212 (32.3)
Ex‐smoker	1387 (40.7)	154 (23.5)
Current smoker	500 (14.7)	290 (44.2)
Occupational risk factor, *n* (%)		
Yes	529 (16.2)	
No	2743 (83.8)	

^a^Age range for discovery cohort: visible haematuria [23–96 years], nonvisible haematuria [23–90 years]. ^b^Age range for the validation cohort: visible haematuria [18–98 years], nonvisible haematuria [25–88 years].

### Development of the haematuria cancer risk score

Spearman's correlation between bladder cancer predictors showed that no strong correlation was observed between predictors (Table S2). Multivariate logistic regression model reports that increasing age (OR 2.9, 95% CI 2.3–3.6, *P* < 0.001), VH (OR 3.9, 95% CI 2.6–5.6, *P* < 0.001), male gender (OR 1.8, 95% CI 1.3–2.4, *P* < 0.001) and smoking history [ex‐smoker (OR 1.5, 95% CI 1.1–2.0) and current smoker (OR 2.6, 95% CI 1.7–3.8), *P* < 0.001] were independently associated with a bladder cancer diagnosis (Table 2). Patients who were ex‐smokers were more at risk compared to current smokers in univariate logistic regression but following adjusting for age in a bivariate logistic regression model and in a multivariate regression model, current smokers were more at risk for bladder cancer. The HCRS was developed as the linear predictor of the fitted multivariate logistic regression:

$$\begin{array}{cc} & \text{Haematuria cancer risk score = 0.055*Age}\\ & \;\text{+ 1.348*Haematuria type + 0.576*Gender}\\ & \;\text{+ 0.413*Ex‐Smoker + 0.943* Current‐Smoker} \end{array}$$

**Table 2:** Univariate and multivariable logistic regression models associated with bladder cancer in the development cohort. *N* = 3539 (bladder cancer = 285)

Predictor	Unit	Univariate		Multivariable	
		IQR‐OR^a^ (95% CI)	LR χ² (d.f., *P*)	IQR‐OR^a^ (95% CI)	Δχ² (d.f., *P*)
Age	years	2.931 (2.377, 3.614)	120.07 (1, <2.2e‐16)	2.892 (2.319, 3.605)	120.07 (1, <2.2e‐16)
Haematuria	Nonvisible	1 (ref)			
	Visible	4.526 (3.127, 6.551)	89.007 (1, <2.2e‐16)	3.850 (2.629, 5.638)	84.119 (1, <2.2e‐16)
Smoker^b^	Nonsmoker	1 (ref)			
	Ex‐smoker	1.917 (1.453, 2.531)		1.512 (1.132, 2.018)	
	Current smoker	1.619 (1.112, 2.357)		2.568 (1.719, 3.836)	
	Missing^b^	1.223 (0.621, 2.410)	22.638 (3, 4.8e‐05)	1.283 (0.636, 2.585)	24.257 (3, 2.2e‐05)
Gender	Female	1 (ref)			
	Male	2.960 (2.196, 3.990)	60.044 (1, 9.3e‐15)	1.779 (1.298, 2.438)	13.812 (1, 2.0e‐04)
Ethnicity^c^	White	1 (ref)			
	Non‐White	0.490 (0.248, 0.967)			NSS
	Missing^b^	0.496 (0.274, 0.896)	11.097 (2, 0.00389)		

CI, confidence interval; IQR, interquartile range; LR, likelihood ratio; NSS, not statistically significant; OR, odds ratios; Δχ^2^, delta chi‐square (degrees of freedom, *P*‐value), terms added sequentially (first to last); χ^2^, chi‐square test (degrees of freedom, *P*‐value).

^a^Interquartile range odds ratios for continuous predictors and simple odds ratios for categorical predictors. Model LR χ² (d.f, *P*) = 242.257 (6, <2.2e‐16). ^b^Fourth category for smoking ‘missing’ was created and compared to nonsmoker category in the logistic regression analysis. ^c^Third category for ethnicity and compared to White category in the logistic regression analysis. Harrell's c‐index = 0.768 (95%CI: 0.741, 0.795).

### Validation of the haematuria cancer risk score

**Figure 1:**
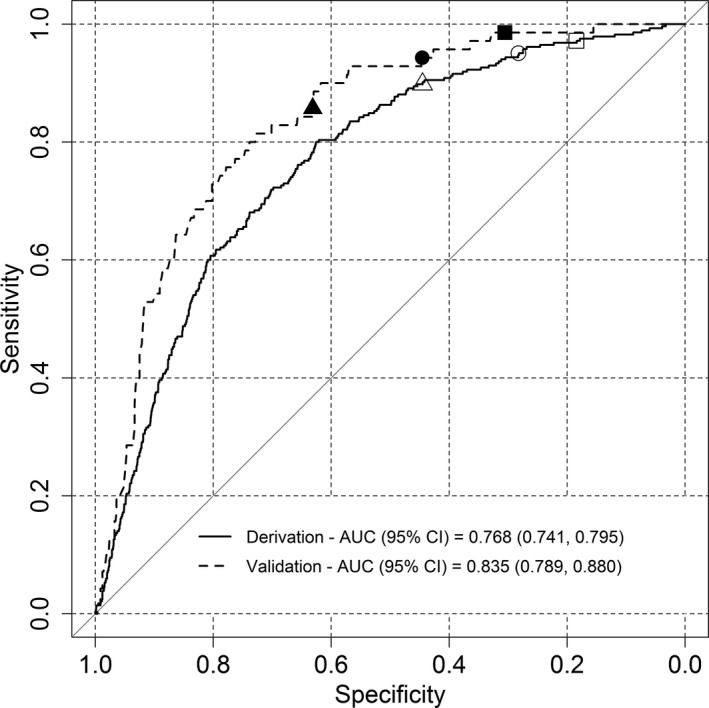
ROC curve of the haematuria cancer risk score. AUC 0.768 (95% CI: 0.741, 0.795) in the development cohort and AUC 0.835 (95% CI: 0.789, 0.880) in the validation cohort. The white square, circle and triangle give 0.972 (95% CI: 0.954, 0.989), 0.951 (95% CI: 0.923, 0.975) and 0.898 (95 %CI: 0.863, 0.930) sensitivity in the development data set with cut‐off values of 4.015, 4.386 and 4.916, respectively. Using the same cut‐off values, the black square, circle and triangle show 0.986 (95% CI: 0.957, 1.000), 0.943 (95% CI: 0.886, 0.986) and 0.857 (95% CI: 0.771, 0.929) sensitivity in the validation data set, respectively.

Figure S2 shows the distribution of the HCRS which was similar between the two data sets. Validation of the HCRS achieves a good discrimination with an AUC = 0.768 (95% CI 0.741–0.795) in the development cohort and AUC = 0.835 (95% CI 0.789–0.880) in the validation cohort (Fig. 1). No statistically significant difference was observed (*P* = 0.1015) between AUC values of the development and validation data sets by Venkatraman's test with 2000 bootstraps 13. The estimated calibration slope in the validation data set was 1.215. The slope is greater than one, but it is not significantly different to one (*P* = 0.151) hence, the discrimination seems to be preserved.

**Figure 2:**
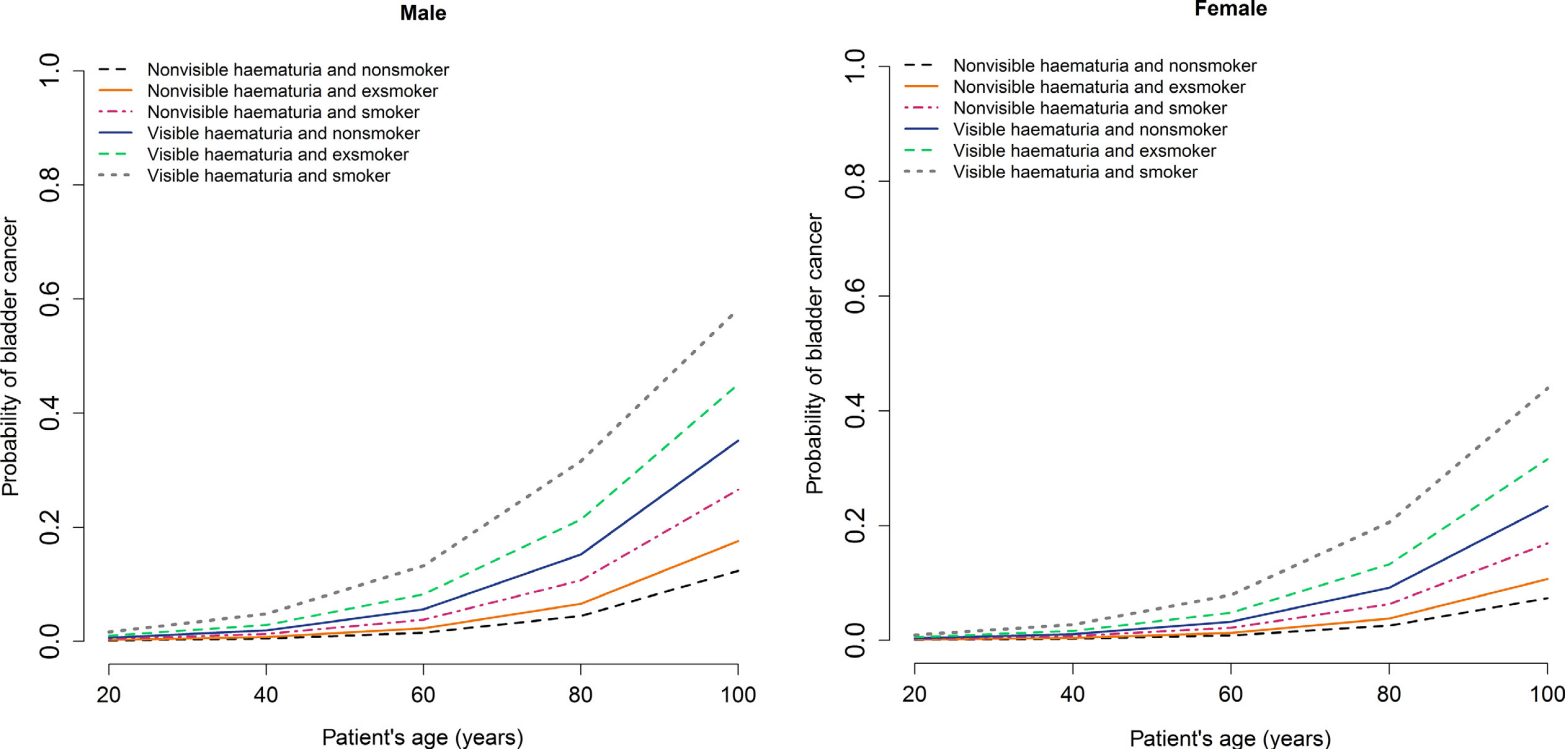
Estimated probability of bladder cancer by age, type of haematuria and smoking history for male (a) and female (b).

Table 3 reports the corresponding sensitivity, specificity, true positives and negatives and false positives and negatives derived from the ROC curve for selected cut‐off values. A bootstrap test with 2000 replicates showed no statistically significant difference between sensitivities of the development and validation cohort (Table S3). Table S4 presents the estimated age cut‐off for NVH and VH patients by smoking status for female and male patients to identify all cancers. Figure 2 illustrates the probability of bladder cancer calculated from the fitted multivariate logistic regression model for male and female patients incorporating other risk factors such as age, type of haematuria and smoking history. Elderly, male smokers with VH had the highest risk of having cancer. Figure S3 shows the haematuria cancer risk score as a nomogram to guide who should be investigated following haematuria.

**Table 3:** Haematuria cancer risk score cut‐offs with corresponding sensitivity, specificity, true positive and negative and false positive and negative derived from the ROC curve in the development and validation cohort

Cut‐off	Development cohort						Validation cohort					
	TP	FN	FP	TN	Specificity (95% CI)	Sensitivity (95% CI)	TP	FN	FP	TN	Specificity (95% CI)	Sensitivity (95% CI)
3.240	282	3	3058	196	0.060 (0.053, 0.069)	0.993 (0.982, 1.000)	70	0	517	69	0.118 (0.092, 0.143)	1.000 (1.000, 1.000)
3.897	279	6	2748	506	0.156 (0.143, 0.168)	0.979 (0.961, 0.993)	69	1	428	158	0.270 (0.232, 0.306)	0.986 (0.957, 1.000)
4.015	277	8	2656	598	0.184 (0.170, 0.198)	0.972 (0.954, 0.989)	69	1	406	180	0.305 (0.268, 0.346)	0.986 (0.957, 1.000)
4.334	274	11	2380	874	0.269 (0.254, 0.284)	0.961 (0.937, 0.982)	67	3	336	250	0.425 (0.386, 0.468)	0.957 (0.914, 1.000)
4.386	271	14	2337	917	0.282 (0.267, 0.298)	0.951 (0.923, 0.975)	66	4	324	262	0.445 (0.406, 0.486)	0.943 (0.886, 0.986)
4.492	268	17	2239	1015	0.312 (0.296, 0.329)	0.940 (0.912, 0.965)	65	5	296	290	0.494 (0.454, 0.536)	0.929 (0.871, 0.986)
4.559	265	20	2171	1083	0.333 (0.317, 0.349)	0.930 (0.898, 0.958)	65	5	285	301	0.512 (0.473, 0.556)	0.929 (0.871, 0.986)
4.681	263	22	2050	1204	0.370 (0.354, 0.387)	0.923 (0.891, 0.951)	65	5	261	325	0.555 (0.514, 0.596)	0.929 (0.871, 0.986)
4.681	262	23	2050	1204	0.370 (0.354, 0.387)	0.919 (0.888, 0.951)	65	5	261	325	0.555 (0.514, 0.596)	0.929 (0.871, 0.986)
4.916	256	29	1806	1448	0.445 (0.428, 0.462)	0.898 (0.863, 0.930)	60	10	216	370	0.631 (0.592, 0.671)	0.857 (0.771, 0.929)

95% CI, 95% confidence interval; FN, false negative; FP, false positive; TN, true negative; TP, true positive.

The development cohort comprised of 55 upper tract cancers (37 renal cell carcinoma and 18 upper tract urothelial cancer [UTUC]) whilst the validation cohort had 12 upper tract cancers (nine renal cell carcinoma and three UTUC). All patients with upper tract cancers would have been selected to have haematuria investigations using the HCRS of >4.5.

### Comparison between haematuria cancer risk score with existing haematuria guidelines

We explored the performance of the HCRS using a defined cut‐off of 4.015, where patients with a HCRS of ≥4.015 should have investigations following a presentation of haematuria. This was based on a sensitivity of approximately 97% for all cancers. We then tested the HCRS in the Switzerland patient cohort. In the external validation cohort, referral for the investigation of haematuria based on NICE guidance would miss 12.9% (*n* = 9) of all urinary tract cancers (six bladder cancers, three renal cell cancers) reporting a sensitivity of 87.1%. Specificity for NICE guidance would equate to 45.7% based on 268 true‐negative cases and 318 false‐positive cases. The AUA recommendation for the investigation of haematuria had a sensitivity of 100% with 80 true‐negative cases and 555 false‐positive cases corresponding to a specificity of 12.6%.

By comparison, using the same HCRS threshold (4.015), a sensitivity of 98.6% was achieved with a corresponding specificity of 30.5% suggesting that an additional 11.4% (*n* = 8) of urinary tract cancers were detected which would have been missed if NICE guidance were applied. The HCRS missed one bladder cancer, a G3 pT1 bladder sarcoma, but reduced the number of patients requiring investigations by 149 patients.

The AUA guidelines for haematuria would identify all cancers but result in a specificity of 3.6% compared to the 30.5% achieved by the risk assessment approach. All patients with upper tract cancers would have been referred for investigation.

## Discussion

This study represents the development and external validation of a HCRS to determine the risk of urinary tract cancer in patients with VH and NVH. We constructed the HCRS using patients from a prospective multicentre observational study allowing generalizability throughout the UK. The score was then validated using a Swiss patient cohort referred for the investigation of haematuria. We show that adopting a risk score approach identified significantly more urinary tract cancers (11.4%) which would otherwise be missed if NICE guidance was applied and reduce the number of patients subjected to investigations compared to AUA guidance.

This study has several strengths in its methodology, patient cohort, ease of use and practical applicability to real‐world clinical practice. We used a reasonable sample size of 3539 patients to derive the HCRS. Our model had a good discriminatory ability in the validation data set with an AUC of 0.835 (95% CI 0.789–0.880) which was higher in comparison with over 60% of prediction risk scores developed by Memorial Sloan Kettering Cancer Centre (MSKCC) which have an AUC of <0.750 15.

The prospective multicentre nature of the development cohort allows for accurate data capture by comparison with most risk prediction scores which are derived from retrospective studies or population data sets 7, 8. External validation using a patient cohort from a different country confirms the risk score is robust and reproducible. Finally, variables chosen represent clinical details which are part of the standard referral criteria for suspected cancer following a presentation of haematuria. Hence, adopting the HCRS would be straightforward without additional time pressures.

Loo and colleagues 16 used electronic medical records (EMR) from Kaiser Permanente to identify patients who had investigations for NVH to derive a development cohort of 1973 patients and a validation cohort of 657 patients. Patients presenting with VH were not accessed. Following multivariable logistic regression, they incorporated the following variables in their Haematuria Risk Index: history of VH <6 months, patient age ≥50 years, smoking history, male gender and >25 RBS/HPF on urine microscopy with a reported AUC of 0.829. The current study which assesses both VH and NVH patients achieves a similar diagnostic performance using fewer variables. Limitations include variable quality of data recorded in EMR and both the development and validation cohorts were derived from the same EMR 17. History of VH within the last 6 months was used as a variable suggesting that these patients were evaluated for VH rather than NVH. Further, some patients did not have complete haematuria investigations and were excluded introducing case selection bias. We could not compare our risk score to that of Loo *et al*. 16 due to the difference in variables recorded.

Another risk score developed by Wu and colleagues was designed to predict the risk of developing bladder cancer based on a case–control study of 678 patients 18. The risk score did not have external validation and incorporated clinical variables such as smoking history and environmental exposure to carcinogens to achieve an AUC of 0.70 (95% CI 0.67–0.73). Incorporating mutagen sensitivity data increased the AUC to 0.80 (95% 0.72–0.82). The risk score by Wu and colleagues was developed to identify patients at risk of developing future bladder cancers who may benefit from screening.

Current recommendations from NICE exclude younger patients (<60 years with NVH and <45 years with VH) for investigation may result in missed cancers 4. Age is the main discriminating factor across guidelines and we show that the use of the HCRS may reduce the risk of missing cancers. In addition to age and type of haematuria, smoking history and gender are important risk factors for the development of bladder cancer 1, 11. These variables are currently not used in the decision to refer for investigations but are collected as part of the standard assessment of patients. We show that incorporating all four variables in a risk assessment approach would improve the patient selection for haematuria investigations compared to current recommendations. The fact that 18% of patients diagnosed with bladder cancer consult their general practitioner ≥3 times prior to referral for investigations suggesting the need for less restrictive recommendations to enable prompt referral for investigations 19.

There are several limitations in this study. The development cohort reflects a UK haematuria referral pattern, and although validated in a Swiss population, further testing in non‐European countries should be considered before use. As discussed previously, recent NICE guidance recommends referral for the investigations of patients with VH aged ≥45 years and ≥60 for patients with NVH. Hence, there may be case selection for patients who were investigated although 16.9% of patients investigated for haematuria were below these age thresholds. Patients were recruited in secondary care, and although guidelines for referral exist to aid primary care decision‐making, it is possible that a case selection bias exists whereby not all patients presenting with haematuria in primary care are referred for investigations according to existing guidelines. The development of a risk assessment tool was not a preplanned analysis; hence, we were limited by the variables we could use. Finally, this study does not attempt to address what are the ideal investigations which should be used for the investigation of haematuria and we have previously attempted to address this in previous studies 20, 21.

## Conclusion

In this study, we report the development and external validation of a risk assessment approach to predict the presence of cancer in patients with VH and NVH. The HCRS improves cancer detection rate and performs better than existing criteria to trigger referral for haematuria investigations. The simplicity of the model with limited clinical variables would facilitate adoption and improve patient and physician decision‐making.

## Ethical approval

The development cohort received approved from the Health Research Authority: North West Liverpool Central Research Ethics Committee on March 2016 (IRAS project ID: 179245, REC reference: 16/NW/0150). The validation cohort was approved by the Cantonal Ethics Committee of Zurich (STV KEK‐ZH BASEC‐Nr. 2016‐00158).

## Conflict of interest

None reported.

## Funding

DETECT I study was funded by the Medical Research Council (MRC) and coordinated by the UCL Surgical Interventional Trials Unit (SITU). John D Kelly is the Chief Investigator for DETECT I. NHS‐associated cost supported all standard of care investigations and procedures. Additional funding was by the UCLH Biomedical Research Centre, The Urology Foundation and The Mason Medical Research Trust.

## Author contributions

Wei Shen Tan had full access to all of the data in the study and takes responsibility for the integrity of the data and the accuracy of the data analysis. All authors had access to statistical reports and tables. Wei Shen Tan, Amar Ahmad and John D Kelly conceived and designed the study. Wei Shen Tan, John D Kelly, Andrew Feber, Hugh Mostafid, Jo Cresswell, Christian D Fankhauser, Sharon Waisbrod, Thomas Hermanns and DETECT I collaborators acquired the data. Wei Shen Tan, Amar Ahmad, John D Kelly, Peter Sasieni, Andrew Feber, Hugh Mostafid, Jo Cresswell, Christian D Fankhauser, Sharon Waisbrod and Thomas Hermanns analysed and interpreted the data. Wei Shen Tan, Amar Ahmad and John D Kelly drafted the manuscript. Peter Sasieni, Andrew Feber, Hugh Mostafid, Jo Cresswell, Christian D Fankhauser, Sharon Waisbrod and Thomas Hermanns made a critical revision of the manuscript for important intellectual content. Amar Ahmad involved in statistical analyses. John D Kelly, Andrew Feber and Wei Shen Tan obtained funding. Wei Shen Tan provided administrative, technical and material support. John D Kelly supervised the study.

## Role of the sponsors

The sponsors had no role in the design and conduct of the study; in the collection, analysis and interpretation of the data; or in the preparation, review or approval of the manuscript.

## DETECT I collaborators

Participating centres and investigators (*principle investigators at each centre): WS Tan, P Khetrapal, H Baker, AN Sridhar, BW Lamb, F Ocampo, H McBain, JD Kelly* (UCLH), K Baillie, K Middleton, J Cresswell, D Watson* (James Cook University Hospital), H Knight, S Maher, A Rane* (East Surrey Hospital), B Pathmanathan, A Harmathova, G Hellawell* (London North West University Healthcare), S Pelluri, J Pati* (Homerton Hospital), A Cossons, C Scott, S Madaan* (Darent Valley Hospital), S Bradfield, N Wakeford, H Mostafid* (Royal Surrey County Hospital) A Dann, J Cook, M Cornwell, R Mills* (Norfolk & Norwich University Hospital) S Thomas, S Reyner, G Vallejera, P Adeniran, S Masood* (Medway Maritime Hospital), N Whotton, K Dent, S Pearson, J Hatton, M Newton, E Heeney, K Green, S Evans, M Rogers* (Northern Lincolnshire & Goole NHS Foundation Trust), K Gupwell, S Whiteley, A Brown, J McGrath* (Royal Devon and Exeter Hospital), N Lunt, P Hill, A Sinclair* (Macclesfield Hospital), A Paredes‐Guerra, B Holbrook, E Ong* (North Devon District Hospital), H Wardle, D Wilson, A Bayles* (University Hospital of North Tees), R Fennelly, M Tribbeck, K Ames, M Davies* (Salisbury District Hospital), JA Taylor, E Edmunds, J Moore* (East Sussex Healthcare NHS Trust), S Mckinley, T Nolan, A Speed, A Tunnicliff, G Fossey, A Williams, M George, I Hutchins, R Einosas, A Richards, A Henderson* (Maidstone Hospital), B Appleby, L Kehoe, L Gladwell, S Drakeley, JA Davies, R Krishnan* (Kent & Canterbury Hospital), H Roberts, C Main, S Jain* (St James's University Hospital), J Dumville, N Wilkinson, J Taylor, F Thomas* (Doncaster Royal Infirmary), K Goulden, C Vinod, E Green* (Salford Royal Hospital), C Waymont, J Rogers, A Grant, V Carter, H Heap, C Lomas, P Cooke* (New Cross Hospital), L Scarratt, T Hodgkiss, D Johnstone, J Johnson, J Allsop, J Rothwell, K Connolly, J Cherian* (The Pennine Acute Hospitals NHS Trust), S Ridgway, M Coulding, H Savill, J Mccormick, M Clark, G Collins* (Tameside General Hospital), K Jewers, S Keith, G Bowen, J Hargreaves, K Riley, S Srirangam* (East Lancashire Hospitals NHS Trust), A Rees, S Williams, S Dukes, A Goffe* (Dorset County Hospital), L Dawson*, R Mistry, J Chadwick, S Cocks, R Hull, A Loftus (Royal Bolton Hospital), Y Baird, S Moore, S Greenslade, J Margalef, I Chadbourn, M Harris, J Hicks* (Western Sussex Hospitals NHS Foundation Trust), P Clitheroe, S Connolly, S Hodgkinson, H Haydock, A Sinclair* (Stepping Hill Hospital), E Storr, L Cogley, S Natale* (Derriford Hospital), W Lovegrove, K Slack, D Nash, K Smith* (King's Mill Hospital), J Walsh, AM Guerdette, M Hill, D Payne* (Kettering General Hospital), B Taylor, E Sinclair, M Perry, M Debbarma* (Pinderfields Hospital), D Hewitt, R Sriram* (University Hospitals Coventry), A Power, J Cannon, L Devereaux, A Thompson* (Royal Albert Edward Infirmary), K Atkinson, L Royle, J Madine, K MacLean* (Royal Cornwall Hospital). R Sarpong, C Brew‐Graves, N Williams (Surgical & Interventional Trials Unit).

## Supporting information


**Figure S1**. Box plot stratifying patients in the development cohort according to the presence of absence of bladder cancer at histology and smoking history according to age.
**Figure S2**. Histogram of the haematuria cancer risk score in the development and validation datasets.
**Figure S3**. Nomogram to guide who should be investigated for cancer following a presentation of haematuria.
**Table S1**. Bladder pathology histology type, grade and stage.
**Table S2**. Spearman's correlation between bladder cancer predictors.
**Table S3**. Comparison of sensitivities of the haematuria cancer risk score in the development and validation datasets based on 2,000 bootstrap replicates for the selected cut‐off values in Table 3.
**Table S4**. Estimated age cut‐off for referral of visible haematuria and non‐visible haematuria to identify all cancers.
