# Supplementary material for: Development and validation of a haematuria cancer risk score to identify patients at risk of harbouring cancer
Source: J Intern Med. 2019 Jan 4;285(4):436–45. doi: 10.1111/joim.12868 (PMC6446724; doi:10.1111/joim.12868)

Appendix

Supplementary Table 1: Bladder pathology histology type, grade and stage.

Supplementary Table 2: Spearman’s correlation between bladder cancer predictors.

Supplementary Table 3: Comparison of sensitivities of the haematuria cancer risk score in the development and validation datasets based on 2,000 bootstrap replicates for the selected cut-off values in Table 3.

Supplementary Table 4: Estimated age cut-off for referral of visible haematuria and non-visible haematuria to identify all cancers.

Supplementary Figure 1: Box plot stratifying patients in the development cohort according to the presence of absence of bladder cancer at histology and smoking history according to age.

Supplementary Figure 2: Histogram of the haematuria cancer risk score in the development and validation datasets. The vertical solid, dashed and dotted lines show the 25th, 50th and 75th centiles of the hematuria cancer risk score in each dataset.

Supplementary Figure 3: Nomogram to guide who should be investigated for cancer following a presentation of haematuria. Each predictor with a given value can be mapped to the Points axis. The sum of these points can be referred to in the Total Points axis.

Appendix

Supplementary Table 1: Bladder pathology histology type, grade and stage.

|  | **Bladder pathology** | **Development cohort, n (%)** | **Validation cohort, n (%)** |
| --- | --- | --- | --- |
| **Bladder pathology histology type** | **Normal** | 3,253 | 586 |
|  | **Bladder urothelial cell carcinoma** | 279 (97.9) | 67 (95.7) |
|  | **Squamous cell carcinoma** | 4 (1.4) | 0 (0) |
|  | **Adenocarcinoma** | 2 (0.7) | 1 (1.4) |
|  | **Sarcoma** | - | 2 (2.9) |
| **Bladder cancer grade** | **G1** | 33 (11.6) | Low grade: 11 (15.9) |
|  | **G2** | 118 (41.4) |  |
|  | **G3** | 134 (47.0) | High grade: 58 (84.1) |
|  | **Not known** | - | 1 |
| **Bladder cancer stage** | **CIS** | 3 (1.1) | 4 (5.7) |
|  | **pTa** | 173 (60.7) | 25 (35.7) |
|  | **pT1** | 57 (20.0) | 21 (30.0) |
|  | **≥pT2** | 52 (18.2) | 20 (28.6) |

Supplementary Table 2: Spearman’s correlation between bladder cancer predictors using pairwise complete observations.

|  | **Age** | **Gender** | **Haematuria** | **Smoking** | **Ethnicity** |
| --- | --- | --- | --- | --- | --- |
| **Age** | 1 | 0.147 | 0.019 | -0.056 | 0.145 |
| **Gender** |  | 1 | 0.280 | 0.09 | -0.026 |
| **Type of haematuria** |  |  | 1 | -0.016 | -0.010 |
| **Smoking status** |  |  |  | 1 | 0.066 |
| **Ethnicity** |  |  |  |  | 1 |

Supplementary Table 3: Comparison of sensitivities of the haematuria cancer risk score in the development and validation datasets based on 2,000 bootstrap replicates for the selected cut-off values in Table 3.

| **Cut-off** | **Development** | **Validation** | **Difference** | **χ²** | **p-value** |
| --- | --- | --- | --- | --- | --- |
| **3.240** | 0.993 | 1.000 | -0.007 | 1.810 | 0.178 |
| **3.897** | 0.979 | 0.986 | -0.007 | 0.710 | 0.400 |
| **4.015** | 0.972 | 0.986 | -0.014 | 2.472 | 0.116 |
| **4.334** | 0.961 | 0.957 | 0.004 | 0.172 | 0.678 |
| **4.386** | 0.951 | 0.943 | 0.008 | 0.442 | 0.506 |
| **4.492** | 0.940 | 0.929 | 0.012 | 0.783 | 0.376 |
| **4.559** | 0.930 | 0.929 | 0.001 | 0.007 | 0.931 |
| **4.681** | 0.923 | 0.929 | -0.006 | 0.144 | 0.704 |
| **4.681** | 0.919 | 0.929 | -0.009 | 0.358 | 0.550 |
| **4.916** | 0.898 | 0.857 | 0.041 | 5.707 | 0.017 |

Supplementary Table 4: Estimated age cut-off for referral of visible haematuria and non-visible haematuria to identify all cancers.

|  | **Female** | | | **Male** | | |  | **NICE** | **AUA** |
| --- | --- | --- | --- | --- | --- | --- | --- | --- | --- |
|  | **Non-Smoker** | **Ex-Smoker** | **Current-Smoker** | **Non-Smoker** | **Ex-Smoker** | **Current-Smoker** |  |
| **Non-visible haematuria** | 73 | 66 | 56 | 63 | 55 | 45 |  | 60 | 35 |
| **Visible haematuria** | 49 | 41 | 31 | 38 | 31 | 21 |  | 45 | - |

NICE: National Institute for Health and Care Excellence, AUA: American Urological Association

Supplementary Figure 1: Box plot stratifying patients in the development cohort according to the presence of absence of bladder cancer at histology and smoking history according to age.


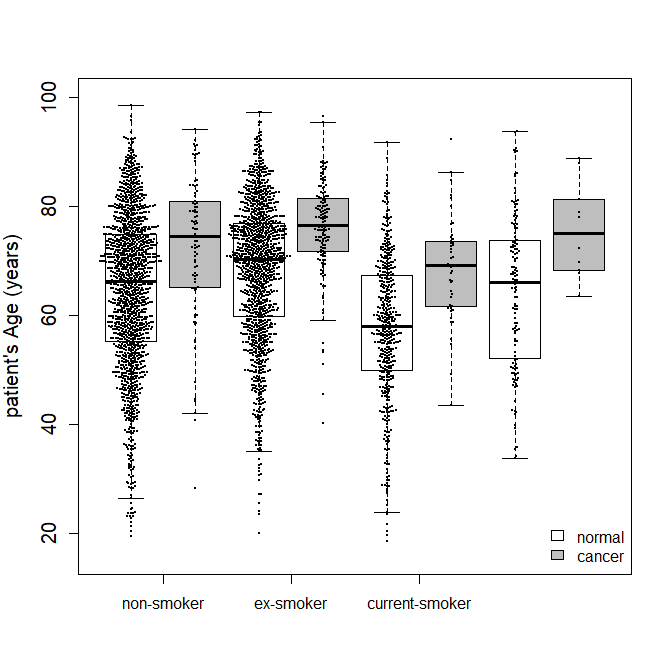


Supplementary Figure 2: Histogram of the haematuria cancer risk score in the development and validation datasets. The vertical solid, dashed and dotted lines show the 25th, 50th and 75th centiles of the haematuria cancer risk score in each dataset.


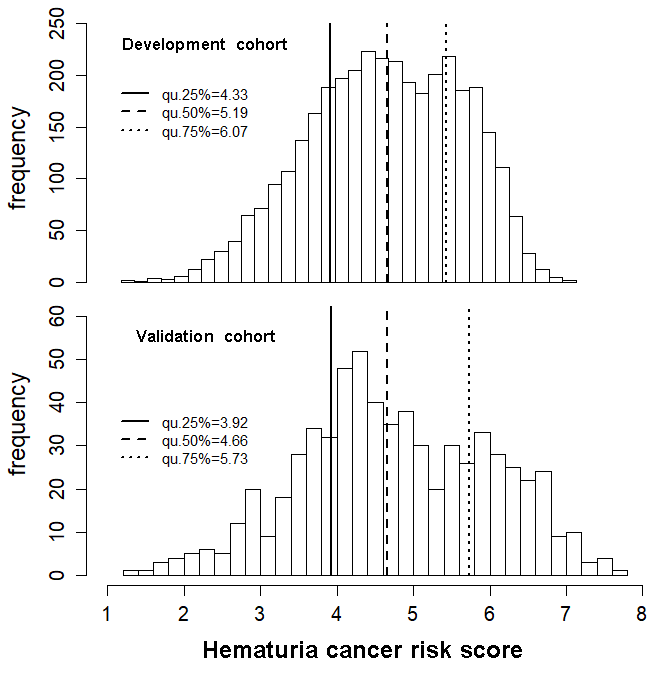


Supplementary Figure 3: Nomogram to guide who should be investigated for cancer following a presentation of haematuria. Each predictor with a given value can be mapped to the Points axis. The sum of these points can be referred to in the Total Points axis.


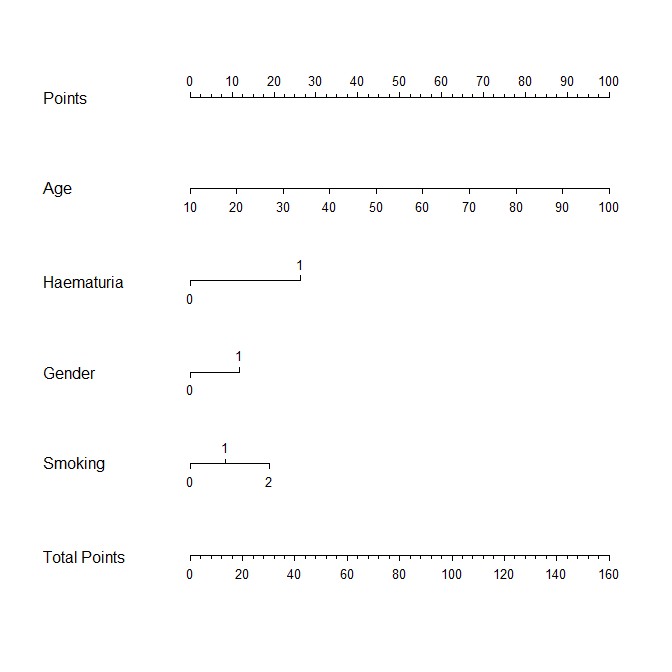

Supplement: Supplementary file 1 — Figure S1. Box plot stratifying patients in the development cohort according to the presence of absence of bladder cancer at histology and smoking history according to age. Figure S2. Histogram of the haematuria cancer risk score in the development and validation datasets. Figure S3. Nomogram to guide who should be investigated for cancer following a presentation of haematuria. Table S1. Bladder pathology histology type, grade and stage. Table S2. Spearman's correlation between bladder cancer predictors. Table S3. Comparison of sensitivities of the haematuria cancer risk score in the development and validation datasets based on 2,000 bootstrap replicates for the selected cut‐off values in Table 3. Table S4. Estimated age cut‐off for referral of visible haematuria and non‐visible haematuria to identify all cancers. [file JOIM-285-436-s001.doc]
